# Detection of macula flava cells proliferation after vocal fold injury

**DOI:** 10.1016/j.bjorl.2026.101915

**Published:** 2026-10-02

**Authors:** Xumao Li, Xiangyu Chen, Sen Yu, Xinsheng Lin, Guangbin Sun

**Affiliations:** Huashan Hospital Fudan University, Department of Otorhinolaryngology-Head and Neck Surgery, Shanghai, China

**Keywords:** Rat, Vocal fold, Injury, Macula flava cell, Proliferation

## Abstract

**Objectives:**

The role of macula flava cells in vocal fold injury repair remains unknown. This study aimed to determine whether they proliferate following vocal fold injury.

**Methods:**

Twenty male Sprague-Dawley rats were divided into control group (n = 5) and experimental group (n = 15). The left vocal fold was injured in rats of the experimental group. The rats were euthanized on postoperative day 1, 3, and 7. Hematoxylin and Eosin (HE) staining was used to examine healing process. Multicolor immunofluorescence was used to detect cell types: proliferating cells (5-Ethynyl-2'-deoxyuridine, EdU), macula flava cells (Glial Fibrillary Acidic Protein, GFAP), and fibroblasts (vimentin).

**Results:**

One day post-injury, the injured vocal fold lacked an epithelial layer and exhibited tissue edema. Three-day post-injury, epithelial cells had begun to cover the injured region. By seven-day post-injury, the injured region fully covered by epithelial cells and displayed atrophy. EdU-positive fibroblasts in the lamina propria significantly increased at day 3, followed by a pronounced decrease at day 7 (p < 0.05). The macula flava cells of injured vocal fold proliferated at day 3 and 7 (p = 0.008).

**Conclusion:**

Vocal fold injuries were repaired within seven days. The results indicated that proliferate behavior differs between macula flava cells and fibroblasts during vocal fold injury repair. Macula flava cells may be also involved in the injury repair.

## Highlights

• The role of macula flava cells in vocal fold injury repair remains unclear.
• Macula flava cells exhibit different biological behaviors from fibroblasts.
• Macula flava cells proliferating day 3 and 7 post-injury.
• Macula flava cells may be involved in the injury repair.

## Introduction

Hoarseness can seriously affect quality of life. According to statistics, one third of the population experiences hoarseness at least once in their lives.^1^ The main cause of hoarseness is vocal fold injury, which includes infections, benign and malignant lesions, surgical trauma, and gastroesophageal reflux.^2^ The healing process of these injuries can be divided into three phases: inflammation, proliferation, and maturation.^3^^,^^4^ Inflammation mainly occurs within 3d after injury, and various cells, including fibroblasts, migrate to the lesions and release a series of cytokines.^5^ Following this, the proliferative phase, which extends over a period of 3- to 4-weeks, is primarily characterized by mesenchymal cell proliferation, epithelialization, and angiogenesis. Finally in the maturation phase of vocal fold injuries, which typically persists for 6- to 12-months,^3^^,^^6^ the primary process involves the remodeling of the extracellular matrix, specifically collagen and elastin.

Vocal fold injury repair involves cell proliferation, migration, and metabolic changes that involve cells such as epithelial cells and fibroblasts. However, whether macula flava cells play a role in the repair of vocal fold injuries remains unknown. The macula flava is a special structural part of the vocal fold composed of highly dense cells and extracellular matrix.^7^^,^^8^ Macula flava cells may be involved in the growth and development of the vocal folds as well as the metabolism of the extracellular matrix, thereby contributing to the maintenance of the normal layered structure of the vocal fold and supporting their phonation function.^7^^,^^9^ In 2006, Tateya conducted a study on cells proliferation after vocal fold injury by performing 5-Bromo-2'-deoxyuridine (BrdU) injection combination with immunofluorescence analysis of cell markers to examine cell proliferation following vocal fold injury.^5^ Their results demonstrated that the macula flava cells did not proliferate following vocal fold injury. However, this study had certain limitations. First, based solely on the expression of vimentin in macula flava cells, they were classified as fibroblasts. Although vimentin is positive for the macula flava cells, the Glial Fibrillary Acidic Protein (GFAP) is a specific marker of the macula flava cells.^10^^,^^11^ Secondly, BrdU-based methods for detecting tissue cell proliferation do not reliably reflect the true state of cell proliferation.

The present study aimed to determine whether macula flava cells proliferate following vocal fold injury. We employed a more sensitive and effective 5-Ethynyl-2'-Deoxyuridine (EdU) cell proliferation assay in combination with multicolor immunofluorescence and comprehensively evaluated the proliferation of various cell types, including macula flava cells, fibroblasts, and epithelial cells.

## Methods

### Animals

The animal experimental protocol was approved by the Animal Care and Ethics Committee of the Department of Laboratory Animal Science, Fudan University (No. 202410026S).

A total of 20 male Sprague–Dawley rats aged 8-weeks were divided into control (n = 5) and experimental (n = 15) groups.

In the control group (n = 5), the vocal folds were not damaged, and EdU (1 mg/mL, 5 mg/kg) was injected intraperitoneally 4 h before sacrifice.

In the experimental group, the left vocal fold was injured. The rats were euthanized on postoperative day 1, 3, and 7. EdU (1 mg/mL, 5 mg/kg) was injected intraperitoneally 4 h before sacrifice.

### Vocal fold injury surgery

The rats were anesthetized via intraperitoneal injection (50 mg/kg ketamine and 1 mg/kg midazolam) and securely positioned on the operating table. The glottic region was exposed using a transoral endoscope. The lamina propria in the middle segment of the left vocal fold was injured using a cold instrument, while the macula flava and right vocal fold were protected from damage. Following injury, laryngeal secretions and bloody exudates were promptly cleared to avoid respiratory obstruction. The respiratory and feeding conditions of the rats were closely monitored after recovery from the anesthesia.

### Tissue preparation and hematoxylin-eosin (HE) staining

The rats were euthanized by an overdose of 0.3% sodium pentobarbital administered via intraperitoneal injection. The larynges were immediately excised and preserved in 4% paraformaldehyde at 4 °C for 24 h, followed by decalcification and paraffin embedding. Consecutive slices (5 μm thick) were made in the horizontal plane (parallel to the vocal fold). The slice was examined using routine HE staining to make sure it contained the complete bilateral vocal folds, including the lamina propria and macula flava. The subsequent adjacent section was prepared for multicolor immunofluorescence.

The slices were examined under a DM1000 LED Microscope (Leica, Germany) and digitalized using the slide scan system KF-PRO-120 (Ningbo Konfoong Bioinformation Tech Co., Ltd., Zhejiang, China) via SlideViewer v1.5.5.2 software (Ningbo Konfoong Bioinformation Tech Co., Ltd.).

### Multicolor immunofluorescence

Multicolor immunofluorescence was used to detect proliferating cells (EdU), macula flava cells (GFAP), and fibroblasts (vimentin).

Immunofluorescence was performed on the 5 μm thick paraformaldehyde-fixed and paraffin-embedded sections of rat larynges. Antigens were retrieved using 10 mM citrate buffer (pH = 6.0). Endogenous peroxidases were inhibited with 5% hydrogen peroxide in methanol for 25 min. Specific binding sites were blocked with 3% bovine serum albumin. Incubation with rabbit monoclonal anti-GFAP primary antibody (ab68428; Abcam, Cambridge, UK; 1:1,000 dilution) was performed overnight at 4 °C followed by detection using antimouse Horseradish Peroxidase (HRP)-conjugated polymer secondary antibodies (Agilent Technologies, CA, USA) for 50 min at room temperature. The HRP reaction was visualized via tyramide signal amplification using Alexa Fluor 520-conjugated tyramides (FP1487001KT; Akoya Biosciences, MA, USA, 1:200 dilution). After incubation with a fresh citrate buffer, the same protocol was applied to the anti-vimentin primary antibody (ab8978; Abcam; 1:1,000 dilution), and the result visualized using Alexa Fluor 620-conjugated tyramide (FP1495001KT; Akoya Biosciences, 1:200 dilution). EdU imaging was performed using a Click-iT™ EdU Imaging Kit (C10086; Thermo Fisher Scientific, MA, USA). Finally, cell nuclei were stained with 4'6-diamidin-2-phenylindol (DAPI; c1002; Beyotime, China). The sections were treated with an autofluorescent quenching solution (G1221; Servicebio, Wuhan, China) and mounted in a fluorescence mounting medium (G1401; Servicebio, Wuhan, China). Slides were digitized using a Panoramic MIDI scanner (3DHISTECH, Hungary).

### Statistical methods

The digitized multicolor immunofluorescence slides were examined, and cell counts were performed using ImageJ (Fiji) software (National Institutes of Health, Bethesda, MD, USA) with the same threshold. The percent of proliferating fibroblasts (EdU and vimentin-positive cells) per section of the lamina propria within the injured area was calculated. The proportions of EdU+/GFAP + cells among total GFAP + macula flava cells in the injured vocal fold were quantified. Data analyses were performed using SPSS Statistics software (version 20.0; IBM Corp, Armonk, NY, USA). The unpaired *t*-test, Mann–Whitney *U*-test, and one-way ANOVA was used; p < 0.05 denoted statistically significant.

## Results

In the experimental group, the left lamina propria of the vocal folds was injured during endoscopy (Fig. 1). All the rats successfully underwent surgery without perioperative mortality or complications.

**Fig. 1:**
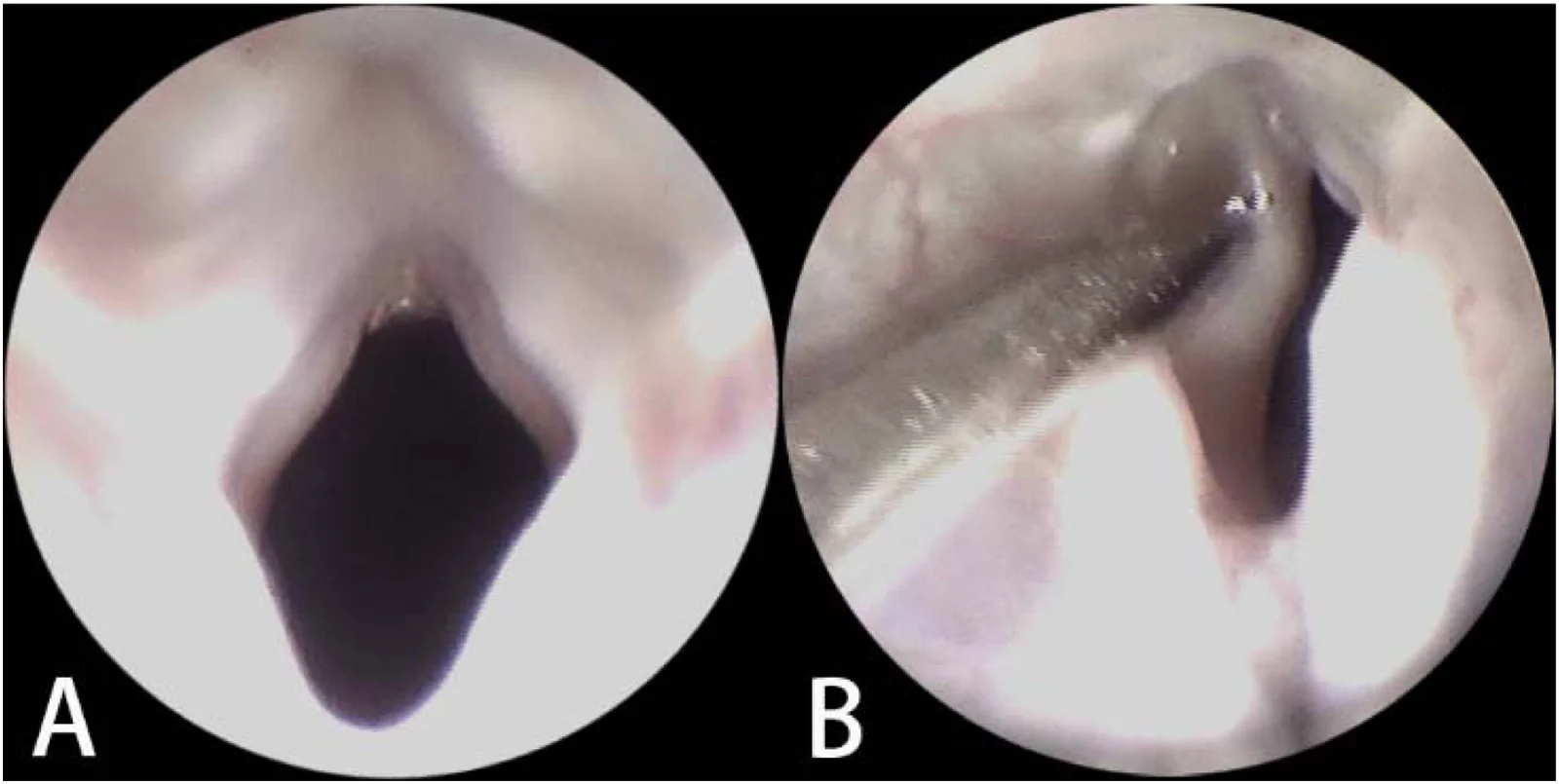
(A) Endoscopic view of the glottic region in a rat. (B) Sharp instrument-induced injury to the lamina propria of left vocal fold.

### HE-staining

One day post-injury, HE-staining revealed the absence of an epithelial layer in the left vocal fold, along with tissue edema (Fig. 2A). Three-day post-injury, the left vocal fold tissue showed marked edema. Epithelial cells covered the wound in the injured region. Cellular proliferation and infiltration significantly increased in the damaged area (Fig. 2B). Seven-day post-injury, HE-staining revealed significant atrophy of the injured region in the left vocal fold. Extensive extracellular matrix deposition was observed in the injured regions. Epithelial cells fully covered the wound surface in the damaged region (Fig. 2C). No damage to the macula flava or right vocal fold was noted in any of the rats.

**Fig. 2:**
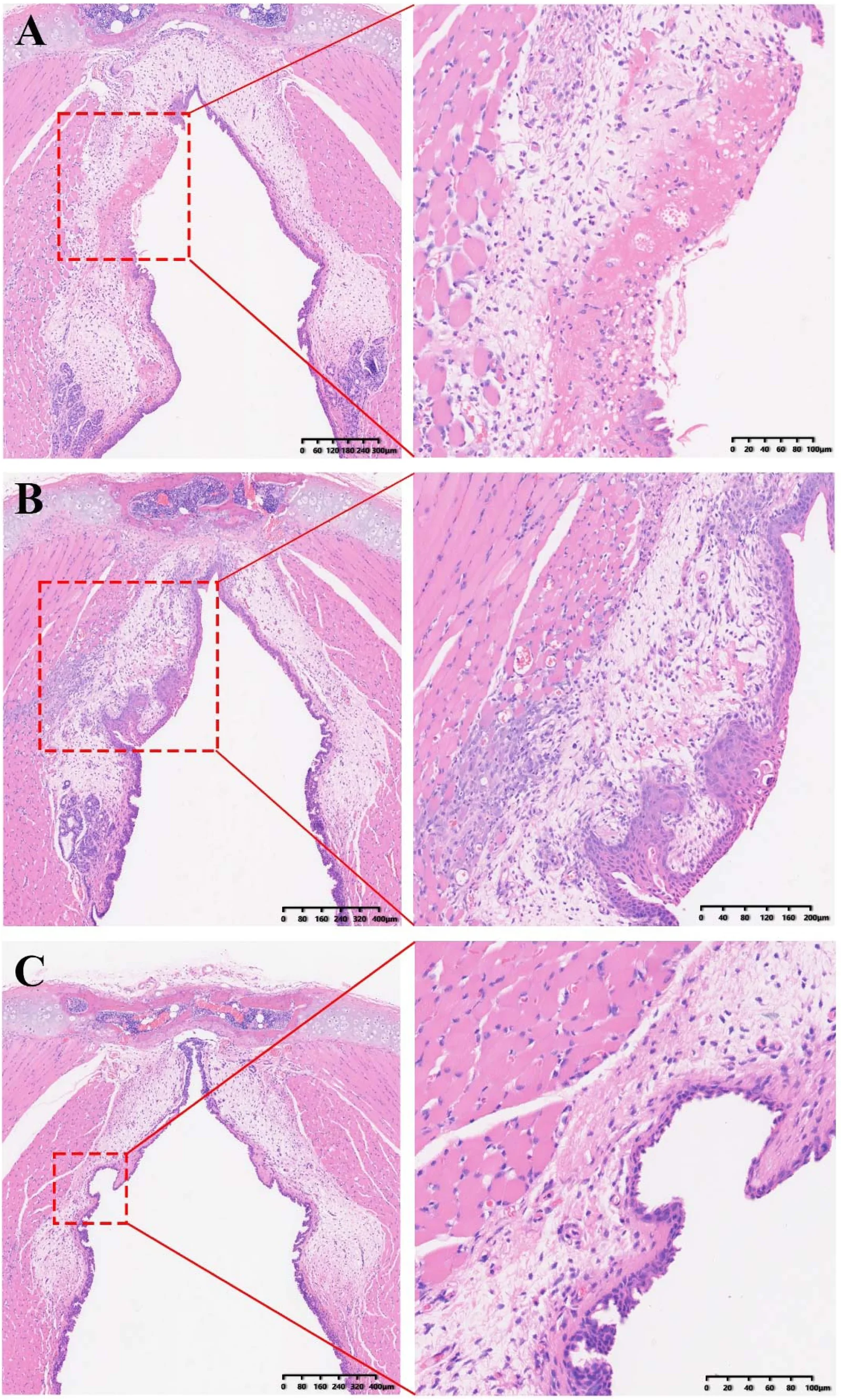
HE-staining of vocal folds following injury: A, B, and C correspond to postoperative day 1, 3, and 7, respectively.

### Multicolor immunofluorescence

In the control group, EdU-positive proliferating cells were scattered in the epithelial layers of both vocal folds. In contrast, no EdU-positive proliferating cells were found in either the lamina propria or the bilateral macula flava (Fig. 3).

**Fig. 3:**
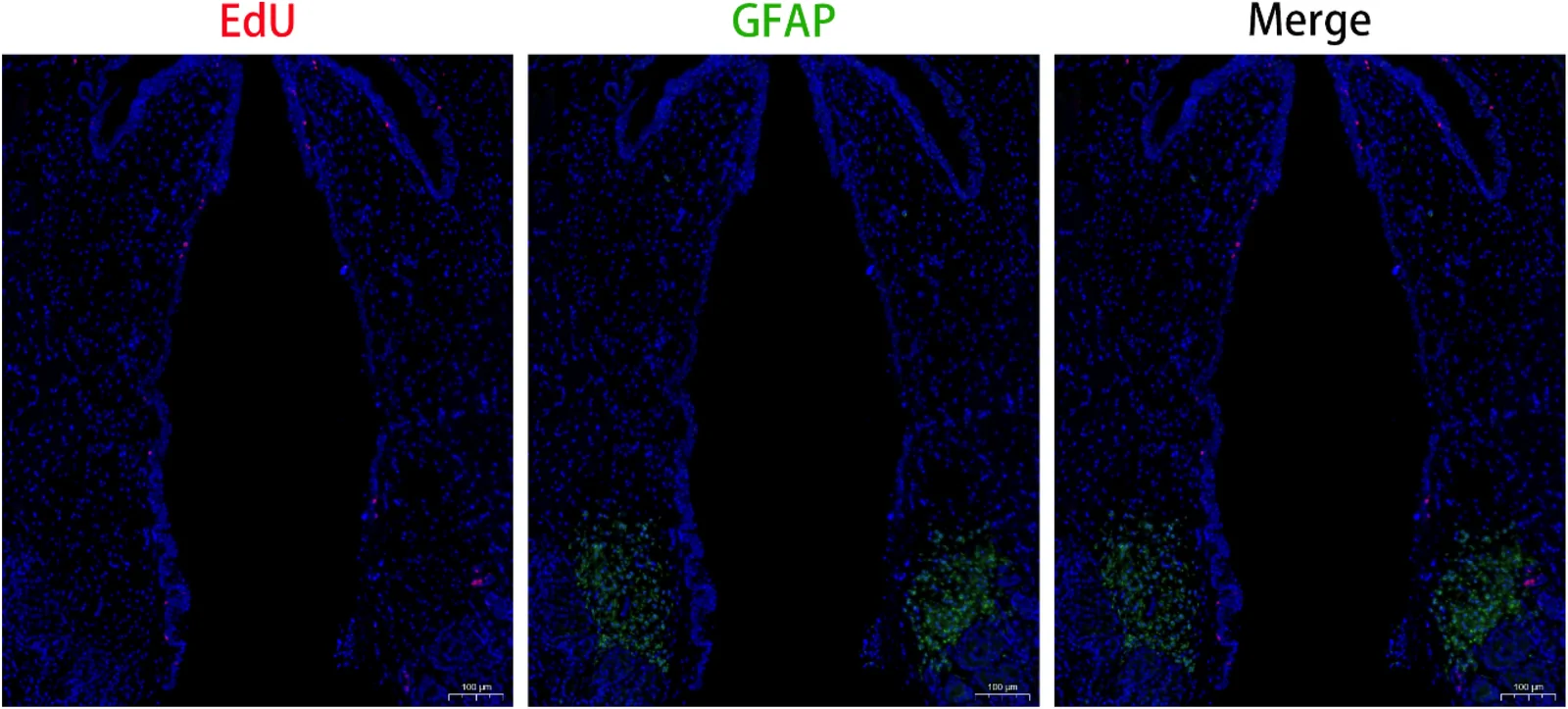
Representative immunofluorescence images of normal vocal fold: Blue stains the nucleus by DAPI; Red is marked with EdU staining; Green is marked with GFAP staining; Merge: DAPI + EdU + GFAP.

### One day post-injury

The injured region of the left vocal fold was distinctly visible and characterized by the absence of an epithelial layer and the aggregation of a substantial number of EdU-positive cells (Fig. 4).

**Fig. 4:**
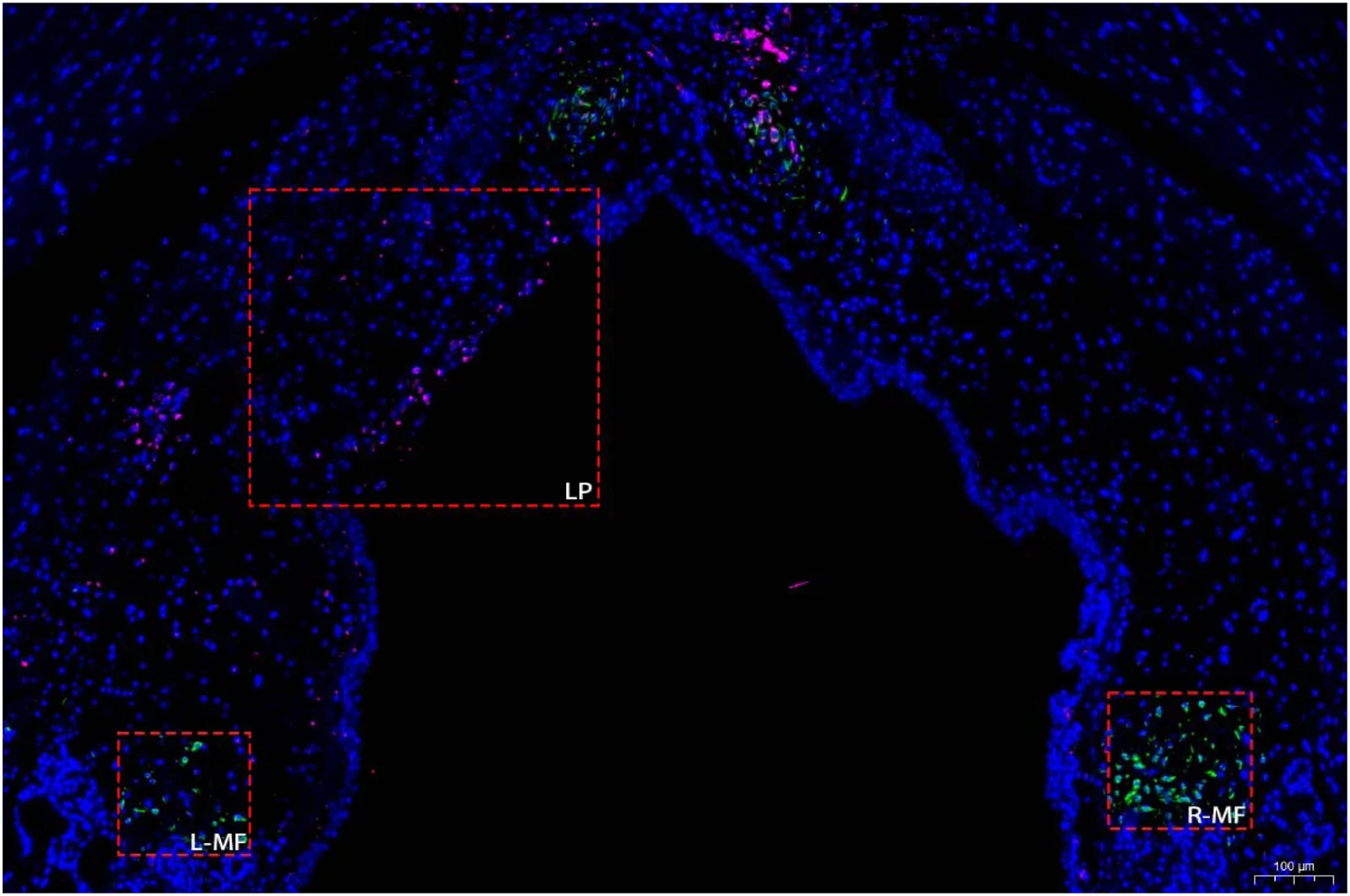
Representative immunofluorescence images of EdU, GFAP, and vimentin of the injured vocal fold day 1 post-injury. LP, Injured Lamina Propria; L-MF, Left Macula Flava; R-MF, Right Macula Flava.

EdU-positive cells were detected in the epithelial layer adjacent to the injured region of the left vocal fold. Within the injured area, vimentin-positive cells (fibroblasts) densely aggregated, with some concurrently showing EdU positivity. In the left macula flava, most cells were positive for GFAP. EdU-positive proliferating cells were scattered around the macula flava but did not co-express GFAP. In the right macula flava, most cells were positive for GFAP and no EdU-positive proliferating cells were detected (Fig. 5).

**Fig. 5:**
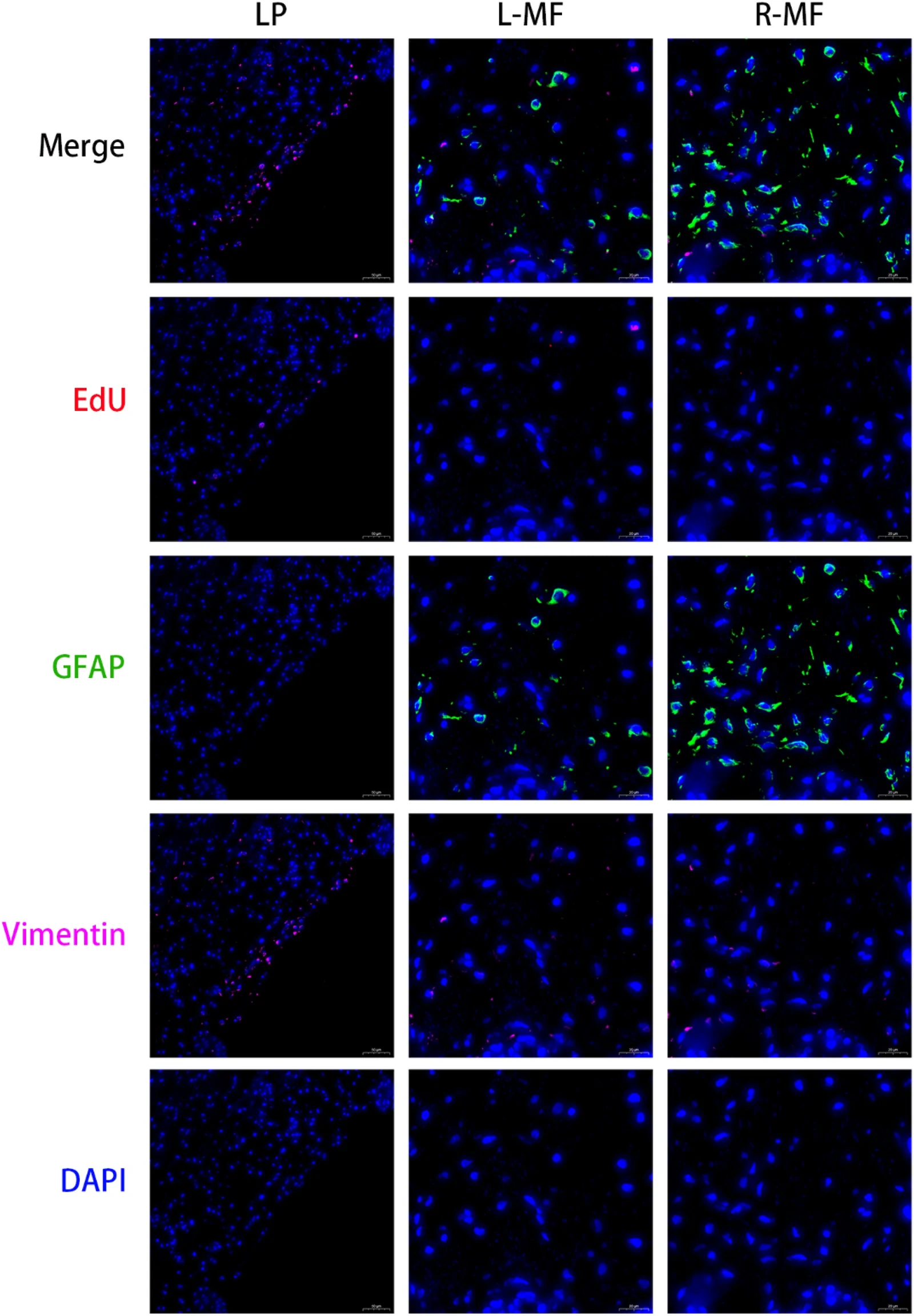
Partial enlarged detail of immunofluorescence day 1 post-injury. LP, Injured Lamina Propria; L-MF, Left Macula Flava; R-MF, Right Macula Flava; Merge, EdU + GFAP + Vimentin + DAPI; Red, EdU; Green, GFAP; Purple, Vimentin; DAPI, Nucleus.

### Three-day post-injury

The injured region of the left vocal fold was distinct. The epithelial layer had reconstituted over the injured area, with a significant aggregation of cells (Fig. 6).

**Fig. 6:**
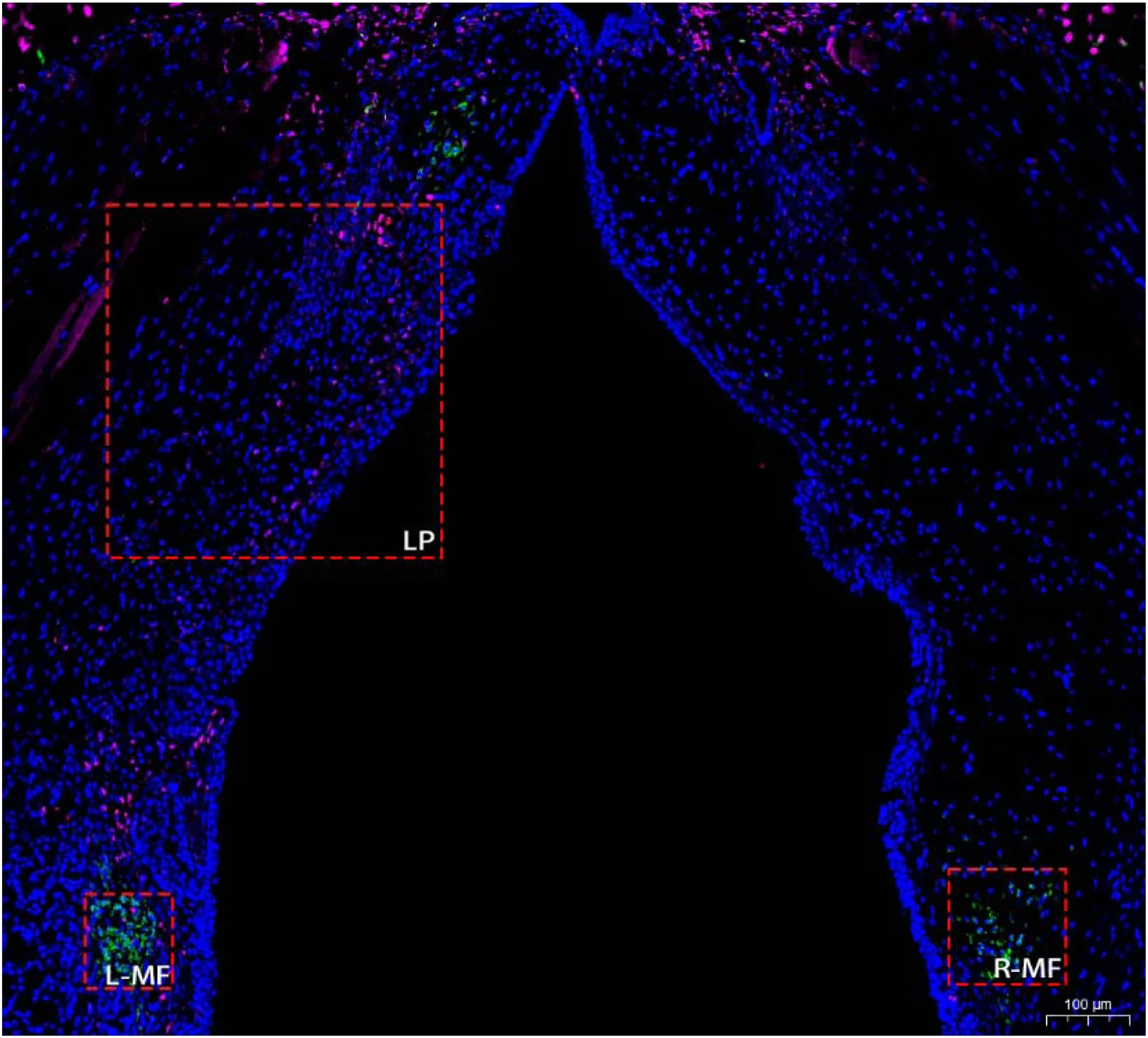
Representative immunofluorescence images of EdU, GFAP, and vimentin of the injured vocal fold day 3 post-injury. LP, Injured Lamina Propria; L-MF, Left Macula Flava; R-MF, Right Macula Flava.

The reconstituted epithelial layer contained scattered EdU-positive cells. A significant number of vimentin-positive cells (fibroblasts) were observed in the injured region, some of which expressed EdU. In the left macula flava, most cells were positive for GFAP and EdU-positive proliferating cells co-expressing GFAP were identified. Conversely, in the right macula flava, most cells were positive for GFAP but no EdU-positive proliferating cells were detected (Fig. 7).

**Fig. 7:**
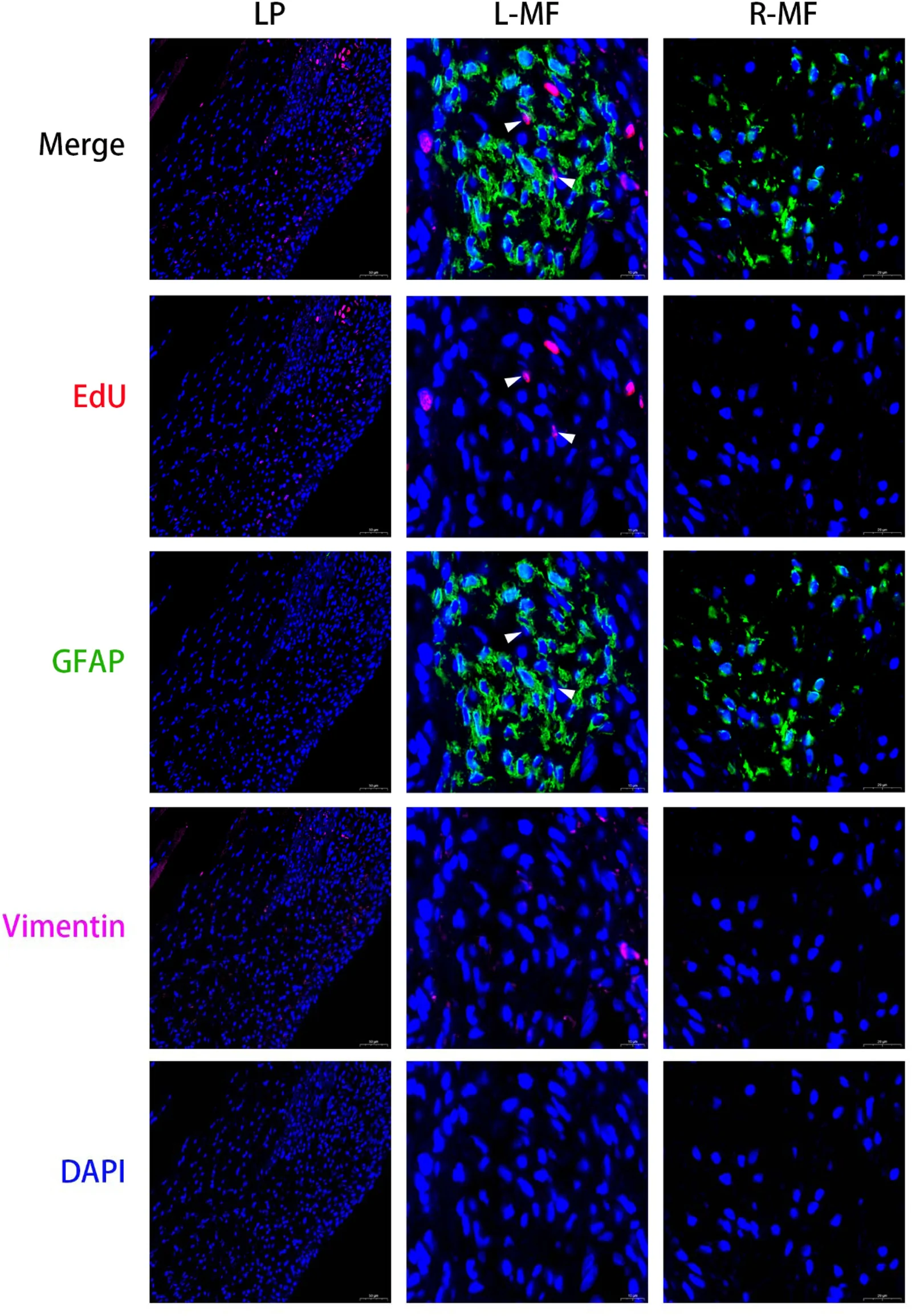
Partial enlarged detail of immunofluorescence day 3 post-injury. Triangles: EdU and GFAP co-expressing cells; LP, Injured Lamina Propria; L-MF, Left Macula Flava; R-MF, Right Macula Flava; Merge, EdU + GFAP + Vimentin + DAPI; Red, EdU; Green, GFAP; Purple, Vimentin; DAPI, nucleus.

### Seven-day post-injury

The injured region of the left vocal fold exhibited marked atrophy. The epithelial layer had fully reconstituted over the injured area, with no significant cellular aggregation (Fig. 8).

**Fig. 8:**
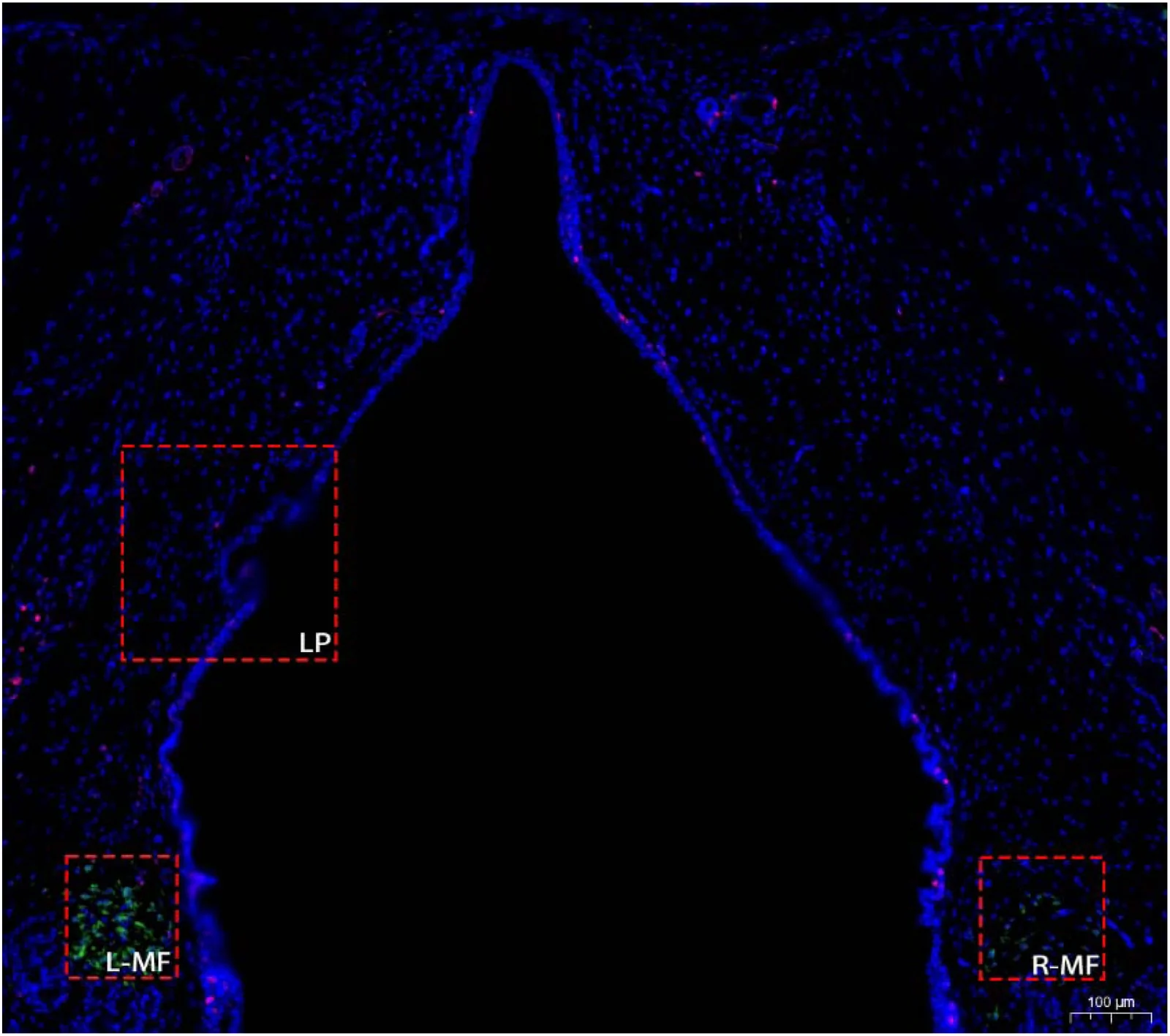
Representative immunofluorescence images of EdU, GFAP, and vimentin in the injured vocal fold day 7 post-injury. LP, Injured Lamina Propria; L-MF, Left Macula Flava; R-MF, Right Macula Flava.

The fully reconstituted epithelial layer contained scattered EdU-positive cells. EdU-negitive, vimentin-positive cells (fibroblasts) were identified in the injured regions. In the left macula flava, most cells were positive for GFAP, and proliferating EdU-positive cells co-expressing GFAP were detected. Conversely, in the right macula flava, most cells were positive for GFAP but no EdU-positive proliferating cells were detected (Fig. 9).

**Fig. 9:**
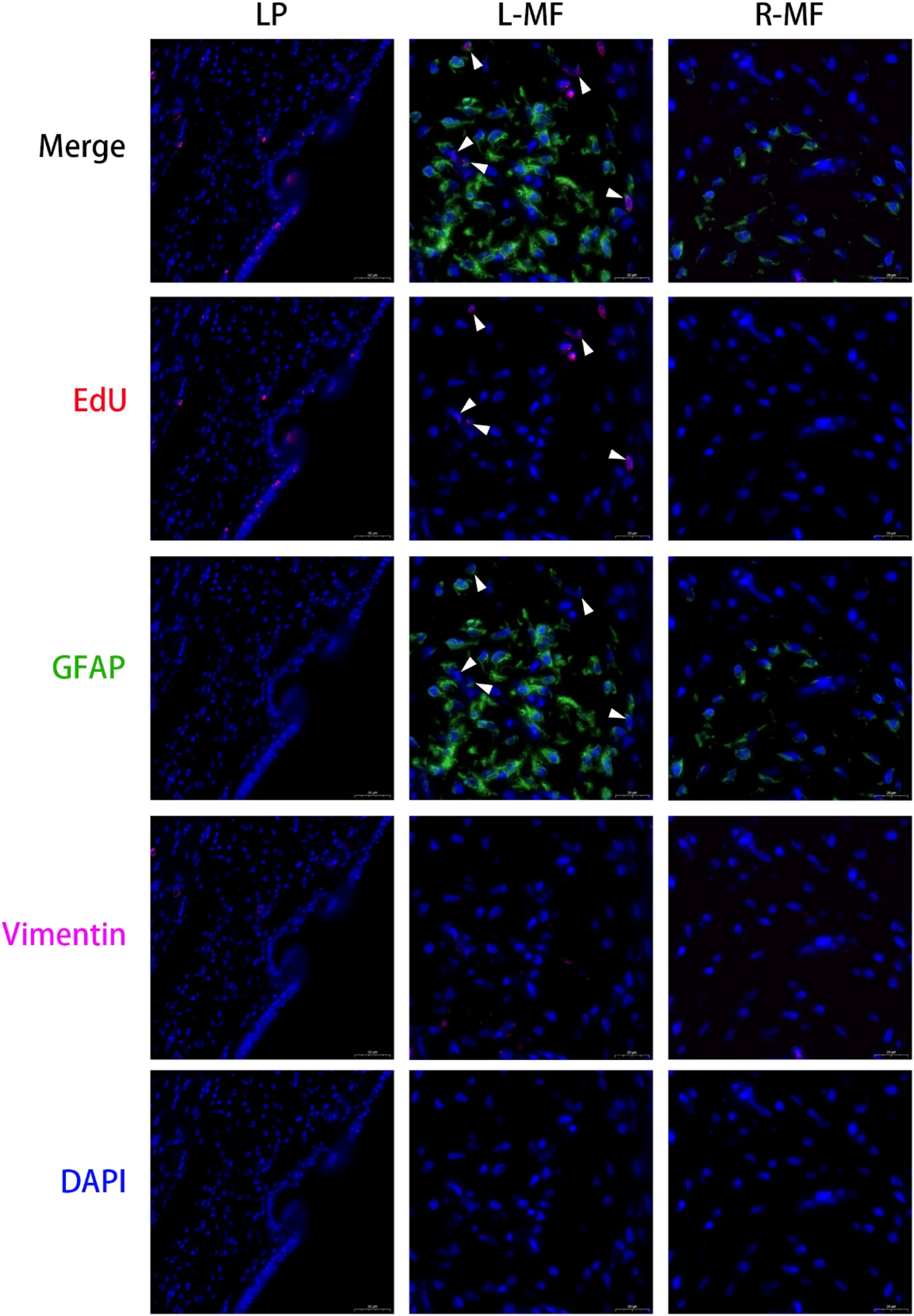
Partial enlarged detail of immunofluorescence day 7 post-injury. Triangles: EdU and GFAP co-expressing cells; LP, Injured Lamina Propria; L-MF, Left Macula Flava; R-MF, Right Macula Flava; Merge, EdU + GFAP + Vimentin + DAPI; Red, EdU; Green, GFAP; Purple, Vimentin; DAPI, nucleus.

Quantitative analysis revealed a significantly increase in EdU-positive fibroblasts in the lamina propria at day 3 post-injury, followed by a pronounced decrease at day 7 (Fig. 10). The proportions of EdU+/GFAP + cells among total GFAP + macula flava cells in the injured vocal fold were quantified as 5.43%±2.49% on day 3 and 8.17%±1.36% on day 7. A statistically significant increase in proliferation was observed in the posterior macula flava of the injured side compared with the contralateral side at days 3 and 7 (p = 0.008). However, no statistically significant difference in proliferation rates was found between days 3 and 7 on the injured side (p = 0.063).

**Fig. 10:**
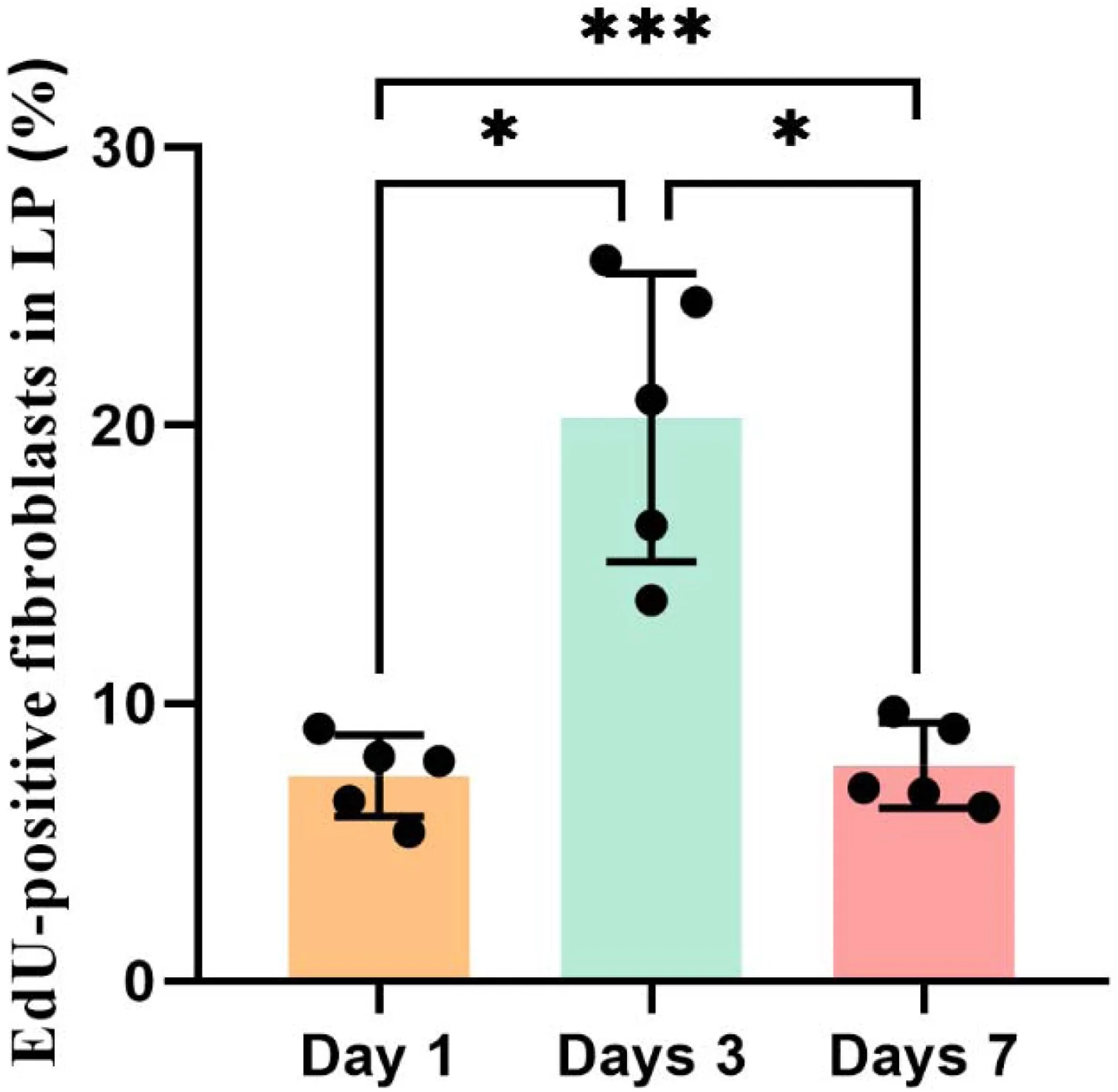
Percent of EdU-positive fibroblasts in the Lamina Propria (LP) after vocal fold injury (* p < 0.05, *** p < 0.001).

## Discussion

In the present study, a rat model of vocal fold injury was established, and the repair process and cell proliferation following injury were dynamically monitored. EdU was administered intraperitoneally before model establishment. Multicolor immunofluorescence analysis was used to evaluate the proliferation of macula flava cells, fibroblasts, and epithelial cells following vocal fold injury.

In the traditional BrdU cell proliferation assay, the high molecular weight of the antibody limits its ability to penetrate cellular membranes, resulting in suboptimal detection efficiency. Consequently, DNA must undergo denaturation pre-treatment typically involving deoxyribonuclease, hydrochloric acid, or heat-induced processing, which disrupt the native DNA structure and consequently reduce the reliability of detection. Furthermore, this assay requires prolonged incubation periods, which complicates the experimental workflow. In contrast, the EdU cell proliferation assay offers superior speed, sensitivity, and accuracy. EdU has a molecular weight approximately 1/500th that of the BrdU antibody, enabling efficient diffusion into cells without requiring DNA denaturation, effectively preventing sample damage during detection. Additionally, EdU detection relies on a chemical reaction rather than antigen-antibody interactions, providing a more precise reflection of DNA replication activity at both the cellular and tissue levels.^12^

Vocal fold injury repair is a complex process involving cellular proliferation, migration, and metabolic changes, wherein epithelial cells and fibroblasts play key roles. As the outermost layer of the vocal folds, the epithelial layer is primarily formed by epithelial cells connected through intercellular junctions, which serve as an essential physical barrier to the vocal folds.^13^ The human vocal fold mucosa is composed of stratified squamous epithelium, which comprises 5–10 layers of non-keratinizing squamous cells^14^^,^^15^ and can rapidly undergo self-renewal to maintain its physiological functions. Experimental findings have demonstrated that renewal of the rat vocal fold epithelium can be completed within 96 h.^16^ Animal studies on vocal fold injury have revealed that epithelial cells rapidly proliferate and migrate following injury^17^^,^^18^ achieving epithelial coverage within day 3–5 post-injury.^7^^,^^8^ In the present study, epithelial cells adjacent to the injured area actively proliferated on 1d after injury and successfully covered the damaged region by the third day, indicating their ability to rapidly proliferate and restore coverage following vocal fold injury.

Fibroblasts are key cellular components within the lamina propria of the vocal folds that are primarily responsible for maintaining the orderly metabolism of the extracellular matrix.^19^^,^^20^ They play a critical role in the repair process following vocal fold injury. They proliferate and secrete collagen while reducing the synthesis of elastin and hyaluronic acid.^5^^,^^21^ In detail, fibroblasts exhibit rapid proliferation and migrate toward the injured site during the inflammatory phase.^5^ In the proliferative phase, the cells undergo significant proliferation.^22^ During the maturation phase, they differentiate into myofibroblasts,^23^ which continuously synthesize and secrete extracellular matrix components to facilitate tissue repair. In this study, fibroblasts were observed in the injured area on the first day after the injury, and their proliferation was more pronounced on the third day. Seven days after the injury, the vocal fold was fully repaired, and no further fibroblast proliferation was detected.

The macula flava of the vocal fold is divided into the anterior and posterior macula flava, which are primarily located at the end of the lamina propria.^7^ The cells in the macula flava are mostly astrocytes that exhibit mesenchymal stem cell characteristics.^24^^,^^25^ The recent studies suggests that hypoxic microenvironment in the macula flava of the human vocal fold promotes the maintenance of macula flava cells stemness.^26^^,^^27^ The current knowledge on the biological functions of macula flava cells, especially during vocal fold injury repair, remains insufficient. This study showed that the biological behavior differs between macula flava cells and fibroblasts during vocal fold injury repair. The macula flava cells in the injured area proliferated on day 3 and day 7. Macula flava cells may be also involved in the injury repair.

A single-pulse EdU labeling approach was employed in this study, with intraperitoneal EdU injection administered 4 h prior to rat euthanasia. This strategy facilitated the labeling of newly proliferated the macula flava cells at distinct time points. In the present study, the proliferation rate of the macula flava cells was relatively low, with values of 5.43%±2.49% on day 3 and 8.17%±1.36% on day 7 post-injury. The previous study demonstrated that the macula flava cells are G0-phase cells.^10^ Our results suggests that the majority of the macula flava cells remain in a stable state after vocal fold injury, while a subset re-enter the cell cycle to further participate in the repair process.

Notably, cumulative labeling was not performed in this study, precluding the assessment of the macula flava cells proliferation rates across different time points. Although the number of newly proliferated the macula flava cells was higher on day 7 than on day 3, this difference did not reach statistical significance, thus limiting the ability to precisely evaluate whether the macula flava cells exert a more prominent function in the later stages of injury repair. In addition, future research should conduct further in-depth investigations on the metabolic alterations of the extracellular matrix of macula flava cells following injury, focusing on the dynamic changes in key components such as type Ⅰ and type Ⅲ collagen, as well as elastin.

## Conclusions

The biological functions of macula flava cells, especially during vocal fold injury repair, remains insufficient. In the present study, the biological behavior differs between macula flava cells and fibroblasts during vocal fold injury repair. The macula flava cells proliferated at day 3 and 7 post-injury. The results indicate that macula flava cells may be involved in the injury repair.

## ORCID ID

Xumao Li: 0000-0001-6019-8109

Xiangyu Chen: 0000-0002-5040-9221

## Ethics committee approval

The animal experimental protocol was approved by the Animal Care and Ethics Committee of the Department of Laboratory Animal Science, Fudan University (nº 202410026S).

## Funding

Project for the Cultivation of Young Talents in Basic Research, Huashan Hospital Fudan University (Grant nº 2024JC001); 10.13039/501100001809National Natural Science Foundation of China (Grant nº 82271154).

## Data availability statement

The authors declare that all data are available in repository.

## Declaration of competing interest

The authors declare no conflicts of interest.
